# Bartholdi’s Colossal “Liberty”

**Published:** 1877-06

**Authors:** 


### *BARTHOLDI'S COLOSSAL "LIBERTY."*

In an article entitled "France to America," in *Scribner* for June, occurs the following description of Bartholdi's Colossal "Liberty," which is to stand in the harbor of New York:

Allowing twenty feet for the height of the island above the water, the pedestal is to be one hundred and ten feet high, and the statue, to the flame of the torch, one hundred and forty-five. This makes the torch at least two hundred and seventy-five feet above the level of the bay. It will equal in height the column in the Place Vendôme at Paris, and will be larger than the colossus of Rhodes, so much celebrated by antiquity. Like that statue, it will have to be cast in pieces of manageable size, and built up much after the manner of an armored frigate. The construction will be a curious piece of engineering skill, for which the sculptor and Mr. de Stuckle will be responsible. At night it is proposed that a halo of jets of light shall radiate from the temples of the enormous goddess, and perhaps the flame of the torch may be fashioned in crystal, in order that it may catch the light of the sun by day, and at night form a glowing object illuminated by electricity.

In respect to the pose of the statue, that has been calculated with care. A Liberty would have to be draped, even if a draped statue were not advisable in a climate so cold as ours, where nude figures suggest extreme discomfort. But M. Bartholdi has also used his drapery to give a tower-like and therefore solid look to his lofty woman, without forgetting the necessity for variety in the upward lines. Or perhaps it would be better to say that he has followed the laws of stability to be seen in the trunks of trees, which are very broad at the ground, where the roots are indicated, yet by no means of one monotonous breadth from the root to the branches.

She will stand so as to suggest that the strongest hurricane could never budge her from the pedestal she has chosen. Her gesture is meant to call the attention of the most distant person, and, moreover, to let him know unmistakably what the figure means. For in this statue, also, M. Bartholdi has applied his science to fine effect in getting the figure outlined against the sky, while the energetic attitude has not interfered with a certain dignified repose which inheres in the resting position, and which may be owing to the weight of the body being thrown on the left leg, as well as to the grave folds of ample drapery. Even if a stranger approaching from the Narrows should not know at once what she is holding up for him to see, the energy of her action will awaken his curiosity, and the dignity of it will make him await a nearer approach with confidence. When he can make out the tablets of the law which jut out from her side as they rest on her bent arm, and the flaming torch which she holds high up above her head, while her eyes are fixed on the horizon, he will be dull indeed if he does not understand what she wishes to tell.

